# AI-Based Classification of Mild Cognitive Impairment and Cognitively Normal Patients

**DOI:** 10.3390/jcm14155261

**Published:** 2025-07-25

**Authors:** Rafail Christodoulou, Giorgos Christofi, Rafael Pitsillos, Reina Ibrahim, Platon Papageorgiou, Sokratis G. Papageorgiou, Evros Vassiliou, Michalis F. Georgiou

**Affiliations:** 1 Department of Radiology, Stanford University School of Medicine, Stanford, CA 94305, USA; rafail99@stanford.edu; 2 Faculty of Electrical Engineering, Mathematics and Computer Science, Delft University of Technology, 2628 Delft, The Netherlands; 3 Department of Neurophysiology, The Cyprus Institute of Neurology and Genetics, Nicosia 2371, Cyprus; rafaelp@cing.ac.cy; 4 Faculty of Medicine, University of Balamand, Balamand 2807, Lebanon; reina.h.ibrahim@std.balamand.edu.lb; 5 2nd Department of Orthopaedic Surgery and Traumatology, Aghia Sophia Pediatric General Hospital, Thivon 3 Street, 15772 Athens, Greece; 6 1st Department of Neurology, Medical School, National and Kapodistrian University of Athens, Eginition Hospital, 15772 Athens, Greece; sokpapa@med.uoa.gr; 7 Department of Biological Sciences, Union, NJ 07083, USA; evassili@kean.edu; 8 Department of Radiology, Division of Nuclear Medicine, University of Miami, Miami, FL 33136, USA; mgeorgiou@med.miami.edu

**Keywords:** mild cognitive impairment, machine learning, Alzheimer’s disease, ensemble model, biomarkers

## Abstract

**Background**: Mild Cognitive Impairment (MCI) represents an intermediate stage between normal cognitive aging and Alzheimer’s Disease (AD). Early and accurate identification of MCI is crucial for implementing interventions that may delay or prevent further cognitive decline. This study aims to develop a machine learning-based model for differentiating between Cognitively Normal (CN) individuals and MCI patients using data from the Alzheimer’s Disease Neuroimaging Initiative (ADNI). **Methods**: An ensemble classification approach was designed by integrating Extra Trees, Random Forest, and Light Gradient Boosting Machine (LightGBM) algorithms. Feature selection emphasized clinically relevant biomarkers, including Amyloid-β 42, phosphorylated tau, diastolic blood pressure, age, and gender. The dataset was split into training and held-out test sets. A probability thresholding strategy was employed to flag uncertain predictions for potential deferral, enhancing model reliability in borderline cases. **Results**: The final ensemble model achieved an accuracy of 83.2%, a recall of 80.2%, and a precision of 86.3% on the independent test set. The probability thresholding mechanism flagged 23.3% of cases as uncertain, allowing the system to abstain from low-confidence predictions. This strategy improved clinical interpretability and minimized the risk of misclassification in ambiguous cases. **Conclusions**: The proposed AI-driven ensemble model demonstrates strong performance in classifying MCI versus CN individuals using multimodal ADNI data. Incorporating a deferral mechanism through uncertainty estimation further enhances the model’s clinical utility. These findings support the integration of machine learning tools into early screening workflows for cognitive impairment.

## 1. Introduction

Mild cognitive Impairment (MCI) can be defined as the in-between stage of dementia and normal aging, reflecting an increased risk of Alzheimer’s Disease (AD). Mild Cognitive Impairment indicates a preclinical phase characterized by mild memory loss that exceeds what is expected from normal aging but does not yet meet the criteria for dementia [1]. The World Health Organization reported in 2021 that 57 million people suffer from dementia, a number expected to triple in the next 30 years [2]. A recent systematic review, which included 66 studies, demonstrated an overall MCI prevalence of around 15%. The same study showed a 10% increase in the prevalence of MCI as the age of individuals progresses from 60 to 80 years of life [3].

The criteria that have to be fulfilled for MCI characterization include complaints of memory impairment by the individual, with intact cognition and no interference of this current state with daily life activities [4]. Although not all MCI cases progress to AD, early identification and monitoring are critical for timely interventions. The required diagnostic evaluation for MCI can be roughly categorized into a subjective and an objective assessment, with the first referring to the history taking, focusing on cognitive function, and the second referring to a set of widely accepted neuropsychological tests; for instance, Mini Mental State Examination (MMSE) [5]. Further investigation for MCI includes cerebrospinal fluid (CSF) analysis, for detection of Aβ1–42 and phosphorylated tau (p-tau) increased levels, reflecting the presence of senile amyloid plaques and neurofibrillary tangles in the brain, and predicting the MCI conversion to Alzheimer’s Disease [6]. In addition to CSF analysis, neuroimaging contributes significantly to MCI detection, with MRI playing a pivotal role in identifying atrophy patterns in key brain regions, revealing candidate biomarkers [7]. These traditional diagnostic methods offer valuable insights but are resource-intensive, expensive, and subject to inter-clinician variability.

The necessity for developing more generalized approaches that provide better reproducibility led to the implication of artificial intelligence (AI) in neurodegenerative disease detection. Machine learning (ML) has evolved the imaging processing and interpretation in dementia research and clinical practice, by facilitating automation and bias reduction in the diagnostic procedure [8]. Regarding ML in disease detection, the models require labeled data for training to accurately identify patterns within novel input data [9]. Several studies utilizing ML models have been developed in recent years [10,11,12]. Deep learning (DL) technology, a subfield of ML, has also been widely explored for MRI and PET-based diagnosis and classification [13,14,15]. DL-based techniques identify patterns distributed across regions of interest, utilizing deep neural networks, without requiring already processed input [16]. The reliance of the DL models on large datasets and the computational complexity required for image analysis limit their applicability in smaller clinical settings. Instead, structured biomarker-based models offer a computationally efficient alternative.

Greater performance and higher levels of accuracy can be achieved by utilizing ensemble techniques instead of individual technologies, providing a fusion of the outcome prediction [17]. Various studies have developed and demonstrated ensemble models for the detection, classification, or prediction of Alzheimer’s Disease, increasing the robustness and accuracy of the established technologies [18,19,20]. These techniques showed great performance; nonetheless, the vast majority of the studies emphasize in neuroimaging application of ensemble models.

Our study aims to develop an ensemble machine learning (ML) model to accurately classify Cognitively Normal (CN) vs. Mild Cognitive Impairment (MCI), using biomarker data from the Alzheimer’s Disease Neuroimaging Initiative (ADNI) database. Cross-validation was conducted for the selection of the best—performing models for the ensemble, and a probability-based classification threshold was introduced to enhance prediction confidence and reduce misclassification errors. This addition has clinical relevance by either lowering the threshold to support earlier detection or raising it to improve recruitment accuracy for clinical studies. Feature variance was estimated in order to identify the most predictive biomarkers for MCI classification, improving both interpretability and clinical applicability of the proposed model. Thereby, the main aims of this work were to design a robust ensemble ML model optimized via cross-validation for early MCI classification, identify the key biomarkers contributing to model predictions, and apply a probability-based threshold to improve classification reliability. Hence, the scope of the study focused on binary classification (MCI vs. CN) using biomarkers from the ADNI dataset, with an emphasis on interpretability and potential clinical translation.

## 2. Materials and Methods

### 2.1. Participants and Data Source

Data used in this study were obtained from the Alzheimer’s Disease Neuroimaging Initiative (ADNI) database (http://adni.loni.usc.edu, accessed on 9 March 2025). The ADNI project was launched in 2003 as a public–private partnership with the primary goal of testing whether clinical, imaging, genetic, and biochemical biomarkers can be combined to measure the progression of Mild Cognitive Impairment (MCI) and early Alzheimer’s Disease (AD). All participants gave written informed consent at the time of enrollment for data collection and sharing. The study protocols and consent forms were approved by the institutional review boards (IRBs) of each participating institution.

For this analysis, we extracted demographic, clinical, and biochemical data from the ADNI repository. Only subjects classified as Cognitively Normal (CN) or any subtype of Mild Cognitive Impairment (MCI, EMCI, LMCI) were included. Subjects diagnosed with Alzheimer’s Disease were excluded to enable binary classification between CN and MCI. No neuroimaging or genetic modalities (e.g., APOE genotype) were used in model development.

Participants diagnosed with early cognitive impairment (EMCI), Mild Cognitive Impairment (MCI), and late MCI (LMCI) were grouped into a single MCI class. Subjects diagnosed with Alzheimer’s Disease (AD) were excluded to restrict the task to binary classification between Cognitively Normal (CN) and MCI individuals.

The dataset contains 3029 entries from the ADNI database, each representing a patient visit record. It includes demographic data (AGE, GENDER), vital signs (VSBPSYS, VSBPDIA, VSPULSE, and VSRESP), biochemical biomarkers (ABETA40, ABETA42, PTAU, TAU, GLUCOSE, PROTEIN, CTWHITE, CTRED, PROTEIN, GLUCOSE, and GENOTYPE), and administrative fields (SUBJECT_ID, NVISIT, VISIT, and SUBJECT_DATE). There is also the RESEARCH_GROUP column, which is for diagnostic labels (CN, MCI, EMCI, LMCI, and AD). Amyloid-β 40 (ABETA40) was excluded due to excessive missingness with over 2100 absent values. Samples with any remaining missing data were then removed, resulting in a filtered dataset containing 1313 MCI cases and 986 CN cases. To balance the classes, 327 MCI samples were randomly removed, producing a final dataset of 1972 subjects that was evenly distributed between CN and MCI groups, 986 subjects per class.

The number of classes after each stage is shown in Table 1.

### 2.2. Feature Selection and Preprocessing

Features retained for analyses included demographic variables such as age and gender, vital signs, systolic blood pressure (VSBPSYS), diastolic blood pressure (VSBPDIA), pulse rate (VSPULSE), and respiratory rate (VSRESP). Moreover, biochemical measurements such as amyloid-β 42 (ABETA42), tau, phosphorylated tau (ptau), CSF protein and glucose, and CSF red blood cells (CTRED) and white blood cells (CTWHITE). Administrative fields such as subject ID, visit number, and visit date were excluded. The APOE genotype variable was also removed to reduce model cost and complexity, as it is one of the most expensive biomarkers.

Gender was encoded as a binary feature, meaning that male subjects were encoded as 1, female subjects as 2, and all continuous variables were scaled to the [0,1] interval using the Min–Max Scaler function from the scikit learn Python (version 3.12.2) library.

Feature variance was calculated to identify the most informative inputs and was used as the selection criterion. This approach assumes that features with the highest variability are more informative for classification tasks. The five features with the highest variance were gender (0.250), age (0.036), diastolic blood pressure (0.020), ABETA42 (0.019), and ptau (0.018). These five features were selected for model development.

A complete summary of variance across features is shown in Table 2.

### 2.3. Model Development

A total of eleven supervised machine learning classifiers were trained using the five selected features: ptgender, age, diastolic blood pressure, ABETA42, and ptau. These models included both traditional and advanced algorithms, such as Naive Bayes, Logistic Regression, K-Nearest Neighbors, Support Vector Machines, Random Forest, Extra Trees, XGBoost, CatBoost, LightGBM, Histogram-Based Gradient Boosting, and a Multi-layer Perceptron. Each model was trained using a stratified 70/30 split and optimized through 5-fold cross-validation with grid search over relevant hyperparameters.

Following cross-validation, the three highest-performing models were Extra Trees, LightGBM, and Random Forest and were selected for final evaluation and integration into an ensemble. These models were chosen due to their superior accuracy when evaluated on the validation set. Three models were chosen (as opposed to two) since this reduces the number of uncertain predictions due to ties in the majority voting process. Notably, they were all tree-based classifiers known for strong performance on tabular data. The ensemble strategy involved majority voting with a probability threshold to improve reliability by abstaining on uncertain predictions.

## 3. Results

### 3.1. Cross-Validation Performance

All models were implemented in Python using the scikit learn library and evaluated on the test set to ensure robustness, accuracy, and generalization. To identify the most promising models, all 11 classifiers were evaluated using stratified five-fold cross-validation on the training set on 70% of the full dataset. Hyperparameter tuning was performed via grid search, and performance was assessed using average validation accuracy across folds.

The results are summarized in Table 3.

These results demonstrate that tree-based ensemble methods consistently outperformed traditional classifiers such as SVM, Naive Bayes, and Logistic Regression. While the Multi-layer Perceptron achieved relatively high best accuracy, its mean accuracy and variance were less stable compared to the top models.

The best performing models based on mean cross-validation accuracy were Extra Trees with an accuracy of 73.2%, Random Forest with an accuracy of 72.3%, and LightGBM with an accuracy of 71.6%. Their respective optimal hyperparameters were: for Extra Trees, max_depth: 18 and n_estimators: 200; for Random Forest, max_depth: None, min_samples_split: 2, and n_estimators: 200; and for LightGBM, learning_rate: 0.1, min_samples_leaf: 2, n_estimators: 100, and num_leaves: 100. These models were selected for subsequent testing on the hold-out validation set.

### 3.2. Evaluation on Hold Out Test Set

To assess model generalizability on unseen data, the three best-performing classifiers from cross-validation, Extra Trees, LightGBM, and Random Forest, were evaluated on the 30% hold-out test set that was not used during training or model selection.

As shown in Table 4, all models performed similarly on the test set, with Extra Trees achieving the highest test accuracy of 0.728 while both LightGBM and Random Forest have accuracies of 0.720. Extra Trees achieved the highest overall performance, with an accuracy of 0.728, an F1 score of 0.736, a precision of 0.757, and a recall of 0.716. The Random Forest model had comparable performance with LightGBM but was a bit worse than Extra Trees.

**Figure 1:**
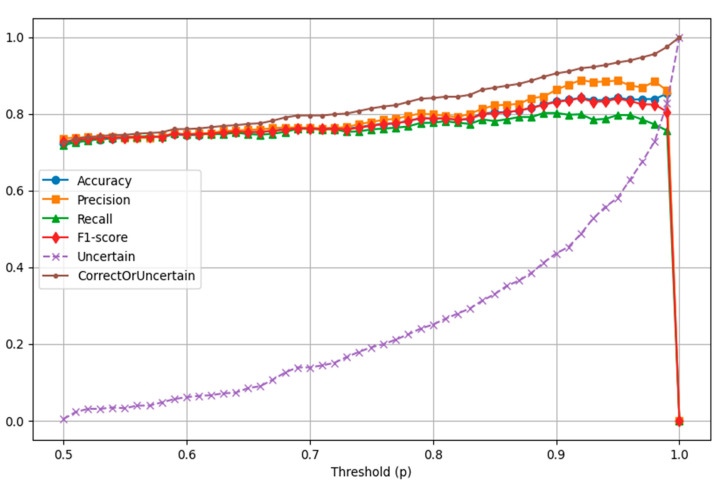
Model performance metrics as a function of voting probability threshold *p* (line graph showing accuracy, precision, recall, and F1 score across varying *p* values).

To improve classification reliability, a majority voting ensemble strategy was implemented. A sample was classified only if at least two of the three base models predicted the same label with an average class probability exceeding a predefined threshold pp. By varying *p* from 0.5 to 1.0, the ensemble allowed flexible control over the balance between precision and recall. As shown in Figure 1, increasing the threshold resulted in higher precision and overall reliability, as the model abstained from making predictions on uncertain cases. However, this came at the cost of reduced recall, since more true positives were left unclassified at higher thresholds.

At a threshold of *p* equal to 0.90, the model achieved its most effective balance between performance and safety, with a precision of 86.3%, a recall of 80.2%, an F1 score of 83.1%, and an abstention rate of 43.6%. Importantly, 90.5% of all predictions were either correct or safely withheld due to low confidence, as shown in Table 5. This approach is particularly beneficial in clinical settings where the consequences of incorrect classification can be significant. By abstaining from ambiguous cases, the model minimizes the risk of false positives or false negatives, allowing those uncertain samples to be flagged for further clinical evaluation. Such a framework supports safer decision-making and can serve as an effective triage tool in diagnostic workflows.

The final ensemble model, integrating the Extra Trees, LightGBM, and Random Forest classifiers, demonstrated improved overall performance by leveraging agreement among high-performing tree-based algorithms. The model only takes into consideration predictions with a probability greater than or equal to *p*, and classifies them only if there is a majority label among those.

This conservative, abstention-aware approach helps mitigate the risk of misclassification, particularly in ambiguous or borderline cases. By deferring low-confidence predictions, the system aligns with clinical needs where incorrect classifications may lead to unnecessary interventions or missed diagnoses. These results underscore the ensemble’s potential as a decision support tool that balances precision with safety in diagnostic workflows. Importantly, the confidence threshold *p* is not fixed, and it can be dynamically adjusted depending on the clinical use case. For instance, in high-risk populations or screening scenarios where sensitivity is critical, a lower threshold may be preferred to reduce missed diagnoses. Conversely, in settings where false positives must be minimized, a higher threshold may be more appropriate. This tunability allows clinicians or healthcare institutions to adapt the model’s behavior to their specific risk tolerance and diagnostic strategy, enhancing its real-world utility.

### 3.3. Comparison with Additional Classifiers

In addition to the ensemble approach, a total of eleven individual machine learning classifiers were trained and evaluated, including tree-based models, boosting methods, and traditional algorithms such as SVM, Naive Bayes, Logistic Regression, and K-Nearest Neighbors, as shown in Table 2. While several models demonstrated competitive accuracy, particularly Extra Trees with an accuracy of 72.47% and LightGBM with an accuracy of 71.96% on the hold-out test set, the ensemble model outperformed all individual classifiers in terms of overall balance across metrics. It achieved a higher F1 score of 78.0% and improved precision by 79.6% while maintaining a solid recall of 76.5%, with the added benefit of a built-in abstention mechanism.

Unlike standalone classifiers, the ensemble approach was able to identify and withhold uncertain predictions, reducing the risk of false positives and negatives in ambiguous cases. This added layer of reliability is particularly valuable in clinical applications, where incorrect classifications can have serious consequences. By combining the strengths of multiple high-performing models and deferring uncertain decisions, the ensemble strategy offers a robust, safety-oriented framework for real-world diagnostic support.

Overall, the ensemble model demonstrated the strongest classification performance while at the same time offering a threshold-based mechanism to minimize confident misclassification in borderline cases and suggest to the physicians that more tests are needed.

## 4. Discussion

This study demonstrates the efficacy of an ensemble AI-based biomarker-driven approach in classifying CN vs. MCI patients. A total of 1972 entries from ADNI were used, equally allocated to 986 MCI patients and 986 CN cases. A significant feature that led to the exclusion of many cases was ABETA40. Multiple variables were subsequently examined to identify the most appropriate features that could be utilized for our ensemble model. Although apolipoprotein E ε4 (APOEε4) allele, located in chromosome 19, has a very strong correlation with disease risk and onset age of “sporadic” Alzheimer’s Disease [21], it was excluded from our model’s features, as the complexity regarding disease causation and prediction risk [22], as well as its high cost, disqualify it as a viable option. Various other factors were examined, ultimately resulting in the five features with the highest variance that indicate significance in the prediction: gender, age, diastolic blood pressure, ABETA42, and ptau. These features have been extensively investigated by scholars, characterizing their contribution to MCI and hence AD development. Women have a higher risk of developing AD, as demonstrated by Azad et al. in a 2007 study [23]. Terry et al, in 1983 identified an increased number of amyloid plaques in individuals above 75 years old [24], proposing an association between AD and advanced age. The detection of ABETA42 in plasma and CSF is shown to be an accurate marker for differentiating AD from other forms of dementia [25], and detection of high levels of Aβ42 and hyperphosphorylated tau protein in CSF is associated with increased load of AD histopathological hallmarks in the brain [26]. Ruiz et al. in 2013 correlated high DBP with Aβ40 levels, thereby linking it to an increased incidence of AD [27]. Consequently, unlike deep learning models requiring large imaging datasets, our model relies on structured biomarker data, making it computationally efficient and accessible for clinical use.

### 4.1. Comparison with Existing Approaches

Our ensemble model is composed of three machine learning technologies, which demonstrated the highest performance when cross-validation was conducted on all eleven classifiers that were proposed. The evaluation of the classifiers showed a 73.91% best accuracy of Extra Trees, 72.17% of Random Forest, and 71.59% accuracy of LightGBM, outperforming traditional models that are most frequently implemented in AI-based disease prediction, like SVM and Logistic Regression. To enhance the classification reliability among CN and MCI patients, a majority voting ensemble was employed, requiring agreement among base models and a minimum confidence threshold (*p*). Hence, by increasing the *p* value, precision and reliability improve, as well as uncertainty reduces. The equilibrium between performance and safety was observed when *p* was set at 0.94. Previous models demonstrated classification accuracies ranging from 70 to 80% for CN vs. MCI detection using MRI features. Some characteristic studies conducted in the last five years are [19,28,29]. Therefore, our biomarker-driven model achieves a comparable accuracy of 78.2% without requiring costly neuroimaging, making it a practical alternative for resource-limited settings.

The studies with the most significant contribution σ to the field have also been described in a valuable meta-analysis performed by Battineni and colleagues in 2024, where 24 studies were included, demonstrating the most powerful tools for detecting AD progression [30]. More recently, Rehman Ur-Zia et al. conducted an informative systematic review on AD prediction using neuroimaging modalities as input for deep learning technology, a subfield of machine learning that utilizes artificial neural networks, able to predict patterns after training with raw, unprocessed data [31]. Both studies highlight the significance and applicability of our work in the context of artificial intelligence in Alzheimer’s Disease prodromal stage classification.

### 4.2. Clinical Implications

A key contribution of our study is the clinical relevance of the proposed model. By integrating uncertainty awareness, this model provides clinicians with interpretable risk assessments, allowing them to prioritize uncertain cases for further evaluation. The implementation of probability-based classification thresholds reduces the risk of misclassification, making the model more reliable for early-stage MCI detection. Two hypothetical scenarios can enhance the clinical relevance of this model, demonstrating its implications in both early detection clinic settings and in research centers. In the first case, a patient who has ambiguous cognitive alterations, mainly focusing on mild memory impairment, a strong family history, and a slight reduction in cognitive scales, arrives in the clinic, and our ensemble model is applied while the MRI is pending. The importance of confidence threshold (*p*) adjustment is that in early detection dementia clinics, the reduction in missed diagnoses is vital for minimizing the burden of dementia; hence, lowering the threshold achieves complete sensitivity. Alternatively, in a research study regarding clinical trials, which requires MCI patients’ recruitment, increasing the confidence threshold (*p*) leads to an elevated specificity in the sample, hence reducing the falsely positive cases. This can also be applied in high-cost intervention settings where avoiding overdiagnosis is crucial. In clinical practice, cases with prediction probabilities near the threshold—flagged as uncertain—could prompt additional assessments, such as follow-up imaging, cognitive testing, and closer clinical monitoring. Integrating this uncertainty-aware approach into diagnostic workflows may support more cautious decision-making and help prioritize resources for borderline cases, aligning with current practices that emphasize risk assessment and individualized follow-up.

### 4.3. Limitations and Future Work

Despite the promising results, certain limitations should be acknowledged. The model lacks external validation, and future studies should test it on an independent dataset to assess its generalizability across different populations. For instance, the ADNI database lacks in incorporating younger individuals, as the age of participants ranges between 55 and 90 years old. This could be limiting in a study examining early stages of dementia, as described in [32]. Additionally, expanding feature selection to include MRI or PET scan biomarkers could further enhance classification performance by incorporating imaging-based insights into cognitive decline. A limitation of our work regarding the selection of the sample has to do with the removal of cases when data was missing, instead of applying imputation techniques. This cleaned our data and maintained the integrity of feature relationships without introducing imputation-related assumptions. However, this technique introduces, to some degree, a selection bias. Various studies have utilized fusion AI-based models to identify patterns and detect dementias at early stages, indicating the significance of multimodal techniques [33,34]. Regarding sensitivity and specificity of the model, false-positive results might emerge and be identified as MCI cases when the confidence threshold is low, and false-negative results, respectively, when the threshold is high. This could be potentially resolved by integrating an expanded feature set. Finally, for broader clinical translation, integrating the model into electronic health record (EHR) systems could enable real-time patient screening and decision support, facilitating early detection and timely intervention in clinical settings.

## 5. Conclusions

This study presents an AI-based classification model for early MCI detection, utilizing a combination of Extra Trees, Random Forest, and LightGBM classifiers with a probability-based thresholding mechanism. The adjustable threshold offers a key advantage over similar techniques, enabling its use across a wide range of settings from routine practice in community healthcare centers to large research hospitals, such as academic medical centers, while maintaining high accuracy. The overall accuracy and precision achieved by the model were 83.2% and 86.3%, respectively, demonstrating its potential for AI-assisted diagnostics. The application of this model aims to facilitate an earlier and more consistent MCI classification, based on routine metrics and biomarkers. Notably, certain limitations related to the subjects’ demographics, including age and ethnicity, slightly restrict the widespread applicability of the study. To build upon these results and further enhance the model’s clinical relevance, future research should focus on external validation—by employing independent cohorts from diverse geographic regions—and multimodal feature integration—by combining imaging, genetic, and clinical data—in order to enhance clinical applicability and reproducibility of the model.

## Figures and Tables

**Table 1:** Number of subjects per diagnostic category at each preprocessing stage.

Raw Data		Grouped—Unbalanced		Grouped—Balanced	
Research Group	Count	Research Group	Count	Research Group	Count
CN	1125	MCI	1313	MCI	986
MCI	750	CN	986	CN	986
EMCI	475				
AD	397				
LMCI	282				

**Table 2:** Feature variance values.

Feature	Variance
PTGENDER	0.2497
AGE	0.0357
VSBPDIA	0.0202
ABETA42	0.0191
PTAU	0.0176
TAU	0.0175
VSBPSYS	0.0163
VSRESP	0.0099
VSPULSE	0.0047
CTRED	0.0067
CTWHITE	0.0035
PROTEIN	0.0030
GLUCOSE	0.0014

**Table 3:** Cross-validation accuracy of all classifiers.

Model	Best Accuracy	Mean Accuracy	Standard Deviation
Extra Trees	0.7319	0.7215	0.0058
Random Forest	0.7232	0.7109	0.0063
LightGBM	0.7159	0.7004	0.0085
CatBoost	0.7145	0.7020	0.0073
Histogram GB	0.7145	0.6922	0.0087
XGBoost	0.7123	0.6964	0.0084
SVM	0.6986	0.6442	0.0436
Multi-layer Perceptron	0.6935	0.6704	0.0134
Logistic Regression	0.6688	0.6673	0.0006
K-Nearest Neighbors	0.6688	0.6581	0.0061
Naive Bayes	0.6645	0.6645	0.0000

**Table 4:** Model performance metrics on the test set.

Metric	Extra Trees	LightGBM	Random Forest
True Positive	224	213	217
True Negative	207	213	209
False Positive	72	83	79
False Negative	89	83	87
Accuracy	0.728	0.720	0.720
Precision	0.757	0.720	0.733
Recall	0.716	0.720	0.714
F1 Score	0.736	0.720	0.723

**Table 5:** Model performance for given thresholds.

Metric	*p* = 0.65	*p* = 0.80	*p* = 0.85	*p* = 0.90	*p* = 0.95
Uncertain	50	148	195	258	344
True Positive	204	174	157	138	102
True Negative	204	176	162	140	107
False Positive	65	44	34	22	13
False Negative	69	50	44	34	26
Accuracy	0.753	0.788	0.804	0.832	0.843
Precision	0.758	0.798	0.822	0.863	0.887
Recall	0.747	0.777	0.781	0.802	0.797
F1 Score	0.753	0.787	0.801	0.831	0.840
Correct or Uncertain	77.3%	84.1%	86.8%	90.5%	93.4%

## Data Availability

Publicly available datasets were analyzed in this study. This data is available from the Alzheimer’s Disease Neuroimaging Initiative (ADNI) at http://adni.loni.usc.edu.

## Abbreviations

The following abbreviations are used in this manuscript:

AD	Alzheimer’s Disease
ADNI	Alzheimer’s Disease Neuroimaging Initiative
AI	Artificial Intelligence
AUC	Area Under the Curve
CN	Cognitively Normal
CSF	Cerebrospinal Fluid
DL	Deep Learning
HER	Electronic Health Record
EMCI	Early Mild Cognitive Impairment
FTP	Flortaucipir
GB	Gradient Boosting
IA-Elc	Immunoassay-Electrochemiluminescence
IP-MS-WashU	Immunoprecipitation–Mass Spectrometry at Washington University
LMCI	Late Mild Cognitive Impairment
LLM	Large Language Model
MCI	Mild Cognitive Impairment
ML	Machine Learning
MRI	Magnetic Resonance Imaging
PET	Positron Emission Tomography
ROI	Region of Interest
SPM	Statistical Parametric Mapping
SVM	Support Vector Machine
SUVR	Standardized Uptake Value Ratio
